# Antibody Banding Patterns on the Enzyme-Linked Immunoelectrotransfer Blot (EITB) Assay Clearly Discriminate Viable Cysticercosis in Naturally Infected Pigs

**DOI:** 10.3390/pathogens13010015

**Published:** 2023-12-23

**Authors:** Gianfranco Arroyo, Andres G. Lescano, Cesar M. Gavidia, Teresa Lopez-Urbina, Miguel Ara-Gomez, Luis A. Gomez-Puerta, Javier A. Bustos, Cesar M. Jayashi, Seth E. O’Neal, Armando E. Gonzalez, Hector H. Garcia, on behalf of the Cysticercosis Working Group in Peru (CWGP)

**Affiliations:** 1 School of Veterinary Medicine, Universidad Nacional Mayor de San Marcos, Lima 15021, Peru; cgavidiac@unmsm.edu.pe (C.M.G.); mlopezu@unmsm.edu.pe (T.L.-U.); marag@unmsm.edu.pe (M.A.-G.); lgomezp@unmsm.edu.pe (L.A.G.-P.); cmjayashi@gmail.com (C.M.J.); agonzalezz@unmsm.edu.pe (A.E.G.); 2 Center for Global Health, Universidad Peruana Cayetano Heredia, Lima 15202, Peru; javier.bustos.p@upch.pe (J.A.B.); oneals@ohsu.edu (S.E.O.); hgarcia1@jhu.edu (H.H.G.); 3 School of Public Health and Administration, Universidad Peruana Cayetano Heredia, Lima 15202, Peru; willy.lescano@upch.pe; 4 Cysticercosis Unit, Instituto Nacional de Ciencias Neurologicas, Lima 15030, Peru; 5 School of Public Health, Oregon Health & Sciences University-Portland State University, Portland, OR 97207, USA

**Keywords:** EITB, antibody banding patterns, porcine cysticercosis, *Taenia solium*, Peru

## Abstract

Enzyme-linked immunoelectrotransfer blot (EITB) detects antibodies against seven *Taenia solium* larvae antigens in three protein families (GP50, T24/42, and 8-kDa) with different structures and functions. EITB banding patterns against these antigens in pigs provide information about the course of infection and may discriminate viable cysticercosis. We analyzed the banding patterns and infection outcomes (presence of viable cysts, degenerated cysts, and any cysts) of 512 rural pigs. Banding patterns were grouped into homogenous classes using latent class analysis, and relationships with infection outcomes were assessed. Four classes were identified: 1 (*n* = 308, EITB-negative or positive for the GP50 family), 2 (*n* = 127, positive for GP50 (GP50 family), GP42-39 and GP24 (T24/42 family), but negative for 8-kDa antigens), 3 (*n* = 22, positive for GP50 and T24/42 antigens (GP42-39 and GP24), as well as to 8-kDa bands GP13, GP14, and GP18, but negative for GP21), and 4 (*n* = 55, positive for GP50 and T24/42 antigens, as well as to 8-kDa antigens GP21 and GP18 in combination). Pigs in classes 3 and 4 were more likely to have viable cysts (72.6% and 96.4%, respectively) than pigs in classes 1 and 2 (0.7% and 27.6%, respectively; *p* < 0.001). The number of infections with any cysts was higher in classes 3 and 4 (77.3% and 98.2%, respectively) and lower in classes 2 and 1 (34.7% and 4.9%, respectively; *p* < 0.001). Pigs with viable cysts represented >90% of pigs with any cysts in classes 3 and 4 (94.1% and 98.2%, respectively), while degenerated cysts were frequent among pigs with any cysts in classes 1, 3, and 2 (86.7%, 47.1%, and 43.2%, respectively; *p* < 0.001). EITB banding patterns strongly correlate with cysticercosis infection status in rural pigs, with classes 3 and 4 being more predictive of viable infections.

## 1. Introduction

*Taenia solium* taeniasis/cysticercosis is a zoonotic disease complex endemic in many low- and middle-income countries where informal pig husbandry and inadequate sanitation coexist [1,2]. The lifecycle of *T. solium* includes humans as definitive hosts of the adult tapeworm (taeniasis) and pigs (as well as humans) as intermediate hosts of the tissue larval phase or cysticercus [3,4]. Neurocysticercosis (NCC), the larval cyst infection of the central nervous system (CNS) is a major cause of epilepsy and other neurological morbidity in humans worldwide and accounts for approximately 30% of epilepsy cases in endemic areas [5,6,7]. On the other hand, porcine cysticercosis negatively impacts the economy of small farmers due to the confiscation of infected pork, although in endemic areas, small farmers are often unaware of the infection of their pigs and communities do not participate in cysticercosis control [8,9,10].

The diagnosis of porcine cysticercosis is crucial for *T. solium* control strategies [11], as pigs with viable cysts are potential reservoirs of future tapeworms for humans. Given the relatively short lifespan of rural pigs, their infection rates often reflect the level of environmental contamination with *T. solium* eggs, and changes in disease prevalence in pigs may provide information in monitoring the success of control programs [12,13]. Due to the strong geographic correlation between foci of cysticercotic pigs and the presence of human tapeworm carriers [14], small-radius clusters around cysticercotic pigs may also serve as an effective tool to identify individuals at high risk of taeniasis for timely diagnosis and treatment [15]. However, a critical limitation is the lack of an affordable diagnostic test with adequate sensitivity and specificity to detect viable cysts in pigs in low-resource settings [16]. Postmortem carcass dissection is the gold standard, but it is not feasible as a routine surveillance approach, in addition to being expensive and time-consuming in field studies [17]. The tongue exam, although highly specific, is only moderately sensitive to severe infections and much less sensitive to mild infections, which tend to be substantially more frequent [18]. Serological tests, targeting either antigen or antibody detection, have been widely used in epidemiological studies of porcine cysticercosis. However, major drawbacks include the poor ability of antibody detection tests to discriminate viable from non-viable infections [19], as well as the low specificity of antigen detection tests due to cross reactivity with *Taenia hydatigena* [20,21,22].

The enzyme-linked immunoelectrotransfer blot (EITB) assay detects antibody bands against seven *T. solium* glycoprotein antigens (GP50, GP42-39, GP24, GP21, GP18, GP14, and GP13) [23] and is the reference serological test for porcine cysticercosis due to its high initially reported levels of sensitivity and specificity (99% and 100%, respectively) [24]. The presence of ≥1 EITB antibody bands has been classically interpreted as an indicator of exposure rather than viable infection [19,25]. The high prevalence of EITB antibody bands in pigs from *T. solium*-endemic areas may arise from pigs with viable infections but may also represent transient responses in pigs exposed but not developing cysts, pigs with degenerated or past infections [26], pigs with maternal antibodies transferred by passive immunity [27], and pigs infected with *T. hydatigena* metacestodes (due to cross reactivity of the GP50 band) [28]. The seven EITB antibody bands reflect responses against three distinct antigen families with defined structures and functions. The GP50 antigen family comprises transmembrane proteins that migrate at the 50 kDa position [29]. The T24/42 antigen family comprises a set of proteins called tetraspanins (proteins involved in cell adhesion and proliferation processes) that migrate at 42–39 kDa and 24 kDa positions [30]. The 8-kDa antigen family comprises small excretory/secretory proteins that migrate at 21, 18, 14, and 13 kDa positions and are eventually also found at the 24 kDa position on EITB [31]. Categorizing positive or negative EITB results using optimal band-number cutoffs (≥3 bands) only moderately improves the ability to predict viable infections [32]. In addition, EITB seroconversion in pigs can occur with diverse patterns of positive bands, which does not occur at random and can provide additional information about the likelihood of viable infection [33].

Unlike humans, the immune response profiles observed in the EITB banding patterns of pigs are influenced by several factors, such as their short lifespan, intensity of exposure, concomitant larval infections, and maternal immunity [34]. In experimentally infected pigs, EITB results correlate with the stage of cyst viability (positive in pigs with viable cysts and negative in pigs without cysts or only with degenerated or caseous cysts) [35], whereas in naturally infected pigs, a proportional trend between the number of EITB bands and cyst burden was found [36]. A previous study also found a correlation between EITB banding patterns and brain imaging findings in NCC patients, and some patterns were found to be highly predictive of viable infections [37]. However, this association has not been properly assessed in naturally infected pigs yet. This paper reports the use of EITB banding patterns for discrimination of viable cysts in naturally infected pigs.

## 2. Materials and Methods

### 2.1. Study Design and Data Sources

This study is a cross-sectional assessment of the relationship between EITB banding patterns and viable cyst infection in naturally infected pigs involving the analysis of data (necropsy outcomes and EITB results) from three studies previously conducted in *T. solium* cysticercosis-endemic rural areas. The first study (year 2006) was a cysticercosis prevalence assessment in 326 pigs of ≥1 months of age from six rural communities in Tumbes, Peru [14]. The second study (year 2012) involved a cohort of pigs that was part of a vaccination trial against porcine cysticercosis in Piura, northern Peru, including only unvaccinated control pigs ≥ 4 months (*n* = 104) who were raised under field conditions [38]. The third study (years 2008–2012) included 83 adult pigs (≥12 months old) purchased from peasants in rural villages of Huancayo in the highlands of Peru (unpublished data, CWGP), considered a highly endemic area for human and porcine cysticercosis [39]. Pigs from Huancayo were consecutively purchased among animals found to be positive upon tongue examination, whereas pigs from Tumbes and Piura were obtained from studies in community settings [14,38].

Pig necropsies were conducted using the procedures extensively applied in previous studies by our group [32,36]. We defined the following infection outcomes separately: (1) presence of any cysts (viable or degenerated), (2) presence of viable cysts, and (3) presence of degenerated cysts. We used these criteria, since pigs with degenerated cysts (with or without viable cysts) may correspond with pigs with long-lived infections. We also obtained the proportion of pigs with viable cysts and degenerated cysts among pigs positive for any cysts. Parasite loads with viable and degenerated cysts were also recorded per pig and included as numerical variables. An EITB assay was performed as previously described by Tsang et al. [23] and adapted by Gonzalez et al. [24]. Banding pattern readings were masked to the infection status of pigs. EITB results are reported as the presence or absence (1 and 0 values) of each of the seven EITB bands. These seven values, together, represent “EITB banding pattern” variable, indicating the combination of the positive bands. The pigs’ sex and age (months) were also registered, and age was categorized as ≤8 and >8 months, as previously described [14,39].

### 2.2. Statistical Analysis

Pig characteristics (age, sex, infection outcomes, and EITB results) were described using summary statistics and compared by study site using bivariate analyses as required. The dichotomous results of each of the seven EITB bands were analyzed using latent class analysis (LCA) to group EITB banding patterns into homogenous groups (“latent classes”) as previously described [37]. We ran different models with increasing numbers of classes (2 to 6), using maximum likelihood methods to select the optimal number of classes according to a consensus between a statistical criterion (the model with the lowest Akaike and Bayesian information criteria (AIC and BIC) for model fit and parsimony, respectively) and the interpretability of classes (representative EITB banding patterns in each class). Pigs with unique EITB banding patterns (representing unusual responses) were excluded from LCA to avoid their inclusion as residuals in latent classes. Classes were described, and class prevalences ≥ 10% and conditional class membership probabilities for each band (close to 0 or 1) were also evaluated to confirm the model structure. Bivariate analyses between EITB classes and characteristics of the pig population were performed. Finally, we used logistic regression models to calculate odds ratios with 95% confidence intervals of viable cysticercosis in pigs according to EITB banding pattern classes and adjusted according to potential confounders (age categories, sex, and degenerated cyst infection). Bivariate models were applied for each covariate of interest. A second model was then applied, including all the covariates that were significant in bivariate analysis. Regression models used clustered-robust variances to account for the correlation of the data by study site. All the analyses were carried out in RStudio v1.2.5001 using the “PoLCA” package for latent class models. Statistical significance in the Wald test was set to 5%.

### 2.3. Ethics Statement

All the information described in this paper was obtained in previous studies by our group and reviewed and approved by The Institutional Ethics Committee for Animal Welfare Ethics of the School of Veterinary Medicine, Universidad Nacional Mayor de San Marcos (approval number 2004-007). All the procedures described in pigs were performed in accordance with the ethical principles outlined by the International Animal Care and Use Committee (IACUC).

## 3. Results

A total of 513 pigs were included (326 (63.5%) from Tumbes, 104 (20.3%) from Piura, and 83 (16.2%) from Huancayo). The median age of the pigs was 9 months (interquartile range (IQR): 5–18 months), although pigs from Huancayo were older than pigs from Piura and Tumbes (*p* < 0.001). Two hundred fifty-three pigs (49.3%) were males, and they were more frequent in Piura than in Huancayo and Tumbes (*p* = 0.027).

Nearly two-thirds of pigs (324, 63.2%) had one or more EITB-positive band. Most pigs from Huancayo were EITB-positive (79/83 (95.2%)), compared to 187/326 pigs (57.4%) from Tumbes and 58/104 pigs (55.8%) from Piura (*p* < 0.001). The median number of EITB bands in pigs was 1 (IQR: 0–3 bands), with higher median values in pigs from Huancayo (median: 6 bands (IQR: 3–7 bands)) than in pigs from Tumbes and Piura (median: 1 band (IQR: 0–2 bands) and median: 1 band (IQR: 0–3 bands), respectively; *p* < 0.001). The most frequent band was GP50 (62.5%), followed by GP42-39 (39.8%) and GP24 (36.8%), whereas low-molecular-weight bands were less frequent (GP21 (10.9%), GP18 (11.5%), GP14 (9.0%), and GP13 (13.8%)).

A total of 130 pigs (25.4%) had either viable or degenerated cysts, 106 pigs (20.7%) had viable cysts, and 55 pigs (10.7%) had degenerated cysts. Pigs with viable cysts represented more than 80% of pigs positive for any cysts (106/130 (81.5%)). Most pigs from Huancayo had viable cysts (76/83 (91.6%)), in contrast to pigs from Piura (12/104 (11.5%)) and Tumbes (18/326 (5.5%), *p* < 0.001), whereas the proportion of pigs with degenerated cysts did not differ between study sites. Pigs with viable cysticercosis had a median of 137 viable cysts (IQR: 49–1000 cysts), and viable cyst burden was higher in pigs from Huancayo than in pigs from Piura and Tumbes (*p* < 0.001). Pigs with degenerated cysts had a median of four cysts (IQR: 1–14 cysts), and the degenerated cyst burden was higher in pigs from Piura than in pigs from Huancayo and Tumbes (*p* < 0.001, Table 1).

Thirteen EITB banding patterns were identified across all studied pigs. One pig had an unusual EITB banding pattern, probably representing a validity issue; therefore, this pig was excluded from LCA. LCA estimations resulted in a four-class model as the optimal model to group EITB banding patterns, with the lowest AIC and BIC compared to models with different numbers of classes (Supplementary Table S1). Each of the four classes presents a distinctive, unique distribution of EITB banding patterns (characteristic of antibody responses against *T. solium* antigen families) not observed in the other class models. Class 1 (*n* = 308 (60.2%), responses with zero or one positive band) was the most common pattern and included EITB-negative pigs and those positive only for the GP50 band (GP50 antigen family). Class 2 (*n* = 127 (24.8%), responses with two to three bands) was the second most frequent and included pigs positive for bands GP50 (GP50 antigen family), GP42-39 and GP24 (T24/42 antigen family), but negative for 8-kDa antigens (GP21, GP18, GP14, and GP13). Class 3 (*n* = 22 (4.3%), responses with four to five bands) included pigs positive for bands GP50, GP42-39, and GP24, as well as 8-kDa bands GP13, GP14, and GP18, but negative for GP21. Class 4 (*n* = 55 (10.7%), responses with five to seven bands) included pigs positive for bands GP50, GP42-39, and GP24, as well as 8-kDa bands GP21 and GP18 in combination (Table 2). Conditional class membership probabilities were very high (≥87%) for bands GP50, GP42-39, and GP24 in classes 2, 3, and 4, respectively, and high (≥78%) for low-molecular-weight bands GP21, GP18, GP14, and GP13 in class 4 (Supplementary Table S2).

The distribution of EITB banding pattern classes statistically differed by study site, with responses of classes 1 and 2 being more frequent in pigs from Tumbes and Piura and responses of classes 3 and 4 more frequent in pigs from Huancayo (*p* < 0.001, Table 3). Older pigs were also found in classes 3 and 4 (*p* < 0.001, Table 3). The presence of viable cysts in pigs statistically differed between EITB classes (*p* < 0.001), as most pigs in class 3 had viable cysts (16/22 (72.7%)), as did nearly all pigs in class 4 (53/55 (96.4%)) compared to class 2 (35/127 (27.6%)), whereas only two pigs in class 1 had viable cysts (Table 3). Infections with any cysts (viable or degenerated cysts) were also more frequent in classes 3 and 4 (17/22 (77.3%) and 54/55 (98.2%), respectively) than in classes 1 and 2 (15/308 (4.9%) and 44/127 (34.7%), respectively; *p* < 0.001). Degenerated cysts were also more frequent in pigs of classes 3 (8/22 (36.4%)) and 4 (15/55 (27.3%)) and less frequent in classes 2 (19/127 (15.0%)) and 1 (13/308 (4.2%), *p* < 0.001). Pigs with viable cysts accounted for most pigs with any cysts in classes 2, 3, and 4 (79.6%, 94.1%, and 98.2%, respectively; *p* < 0.001), whereas pigs with degenerated cysts were more frequent among pigs with any cysts in class 1 (86.7%, *p* < 0.001). Viable cyst burden per pig was also higher in classes 3 and 4 and lower in classes 1 and 2 (*p* < 0.001). Degenerated cyst burden was borderline statistically different between EITB classes (Table 3).

The relationship between viable cyst infection in pigs and EITB classes remained strong in the multivariate regression analysis after adjustment by age categories and the presence of degenerated cysts (Table 4). There was approximately fivefold increased odds of having viable cysts in pigs with class 3 responses (OR = 5.63 (95% CI: 1.20 to 26.41), *p* = 0.028), and much higher odds in pigs with class 4 responses (OR = 47.99 (95% CI: 6.75 to 341.01), *p* < 0.001) compared to pigs with class 2 responses (reference level), whereas pigs with class 1 responses had very low odds of having viable cysts (OR = 0.02 (95% CI: 0.00 to 0.14), *p* < 0.001). The odds of having viable cysts in pigs was also statistically higher for pigs with class 4 responses versus pigs with class 3 responses (reference level, OR = 8.52 (95% CI: 5.61 to 12.92), *p* = 0.001). Pigs older than 8 months also showed higher odds of having viable cysts (OR: 6.03 (95% CI: 3.26 to 11.18), *p* < 0.001), while the presence of degenerated cysts was not correlated with statistically higher odds of having viable cysts in the multivariate model (Table 4).

## 4. Discussion

The diagnosis of porcine cysticercosis is challenging because of the difficulty and limitations of current diagnostic options that can be used in endemic settings [16,40]. Necropsies are impractical in field studies, the tongue test is only moderately sensitive, and serological tests for antigen or antibody detection have low specificity due to cross reactions with other cestode infections [17,22] or cannot differentiate between infection and exposure [19]. Our results demonstrate that the heterogeneity of EITB results can be grouped into four distinct EITB banding pattern classes, and among them, classes 3 and 4 are highly predictive of viable cyst infection (>75%), whereas less than 30% of pigs in class 2 had viable cyst infections, and an even lower proportion of pigs in class 1 had viable cysts. EITB banding patterns can substantially improve the discrimination of viable cysts in porcine populations.

A higher proportion of pigs with EITB banding pattern classes 3 and 4 had viable cysts and also had high cyst loads. These classes involved responses to GP50 and T24/42 antigens but also included responses to 8-kDa antigens that correlate with multiple viable parenchymal NCC and subarachnoid NCC in humans [37]. The higher predictive value for viable swine cysticercosis of EITB classes 3 and 4 compared to class 2 responses can be explained by the presence of 8-kDa bands. These antigens are classified as excretory/secretory proteins [31,41], and therefore, the higher the number of viable cysts in pigs, the greater the production of circulating antigens and the more seroconversion, as reflected in class 3 and class 4 EITB responses.

Pigs with class 1 EITB banding patterns had an almost complete absence of viable infections, as only 2/308 pigs had viable cysts. Similarly, the presence of degenerated cysts in this class was lower (less than 5%) compared with classes 2, 3, and 4. A previous study showed that only 2/13 sera from human cases with a confirmed diagnosis of cysticercosis reacted to the GP50 band alone on EITB (false-positive rate of 84.6%) [42], whereas in a study of pigs experimentally infected with *T. hydatigena*, the presence of the GP50 band alone indicated cross reactivity against *T. hydatigena* metacestode antigens [28], highlighting the limited specificity of the GP50 band alone in rural pigs (which may be exposed to a higher number of parasites).

Viable cysts were observed in less than 30% of pigs with class 2 responses, and degenerated cyst infections represented 43.2% of pigs with any cysts in this class, reflecting the persistence of antibody responses to antigens GP42-39 and GP24, which are frequent in class 2 (T24/42 antigen family) [23,43], which may explain the presence of antibodies to these antigens in pigs with degenerated or resolved infections [34]. However, some class 2 pigs with three bands (GP50, GP42-39, and GP24) had a high cyst burden. It is possible that these pigs may also express 8-kDa antigens, which can also migrate to the GP24 band on EITB [31]. In any case, responses of class 2 are not highly predictive of viable infection and should also be considered with caution for diagnosis of viable cysticercosis in naturally infected pigs.

We also observed that pigs older than 8 months had greater odds of having viable cysts in the regression model, and EITB class 3 and 4 responses were also more frequent in pigs older than 8 months. These results are consistent with previous studies [36] and suggest that older pigs have been more exposed to *T. solium* eggs through their lifetime, with more time to develop cysts and a stronger antibody response than younger pigs [39,44,45].

In our study, LCA identified four EITB banding patterns that reflected characteristic responses against *T. solium* antigen families. Class 1 includes negative pigs and responses to GP50 antigens alone, while class 2 includes responses to T24/42 antigens, and classes 3 and 4 include responses to GP50, T24/42, and 8-kDa antigens. EITB classes 1 and 2 were more frequent in the total pig population, since GP50, GP42-39, and GP24 antigens belonging to these classes are immunodominant and apparently induce a more persistent immune response [23,46]. Classes 3 and 4 are less common in the pig population [39]. Classes 3 and 4 showed a similar structure, with both classes including responses to 8-kDa antigens. However, LCA recognizes them as distinct, separate classes that present different clinical and a somewhat different sex/age profiles. Pigs with class 4 responses were also more frequent in Huancayo, where pigs were selected using a tongue test, while EITB classes 1 and 2 were more frequent in pigs from community studies in Tumbes and Piura.

Our findings should be interpreted in light of certain potential drawbacks. We did not determine potential confounders such as the presence of other cestode infections (e.g., *T. hydatigena*), the inclusion of which in the regression model could have provided a more precise estimate of the association between EITB banding patterns and infection outcomes. The use of data from three studies with differences regarding EITB results and infection outcomes may affect the generalizability of our findings. For this reason, we used clustered robust variances that account the correlation of the data by study site in the regression model. Finally, the use of latent classes as predictors in the logistic regression model results in a certain degree of uncertainty associated with a measurement error in the predictor variable, which can produce a bias in the estimation of the regression parameters. Finally, band pattern readings are qualitative rather than quantitative and depend on the expertise of readers, so further studies using quantitative methods, such as Luminex, should more precisely assess the correlation between EITB responses and infection status in porcine cysticercosis.

## 5. Conclusions

The accurate diagnosis of porcine cysticercosis, the intermediate reservoir of *T. solium* infections, is a key component for the effectiveness of control strategies. EITB banding patterns correlate strongly with cyst infection status in pigs, and their interpretation improves the diagnosis of cysticercosis in porcine populations. EITB class 3 and class 4 responses are highly predictive of viable cyst infections, whereas class 2 responses are associated with a combination of viable and non-viable cyst infections, and class 1 responses usually represent pigs without cysts. Even if EITB serves as the reference serological test for porcine cysticercosis, its implementation in rural settings is difficult and not affordable. New, easily applicable assays are required, and the availability of recombinant antigens produced from native antigens in rapid assays (for example, using antigens with greater predictive value and their respective banding patterns) may improve the real-time diagnosis of porcine cysticercosis in *T. solium*-endemic areas.

## Figures and Tables

**Table 1:** Characteristics of the total pig population and distribution by study site.

Characteristic	Total (N = 513)	Study Site			*p*
		Tumbes (*n* = 326)	Huancayo (*n* = 83)	Piura (*n* = 104)	
Age (months)					
Median (IQR)	9 (13)	7 (15)	24 (24)	8 (3)	0.001
≤8 months	237 (46.2)	176 (54.0)	8 (9.6)	53 (51.0)	0.001
>8 months	276 (53.8)	150 (46.0)	75 (90.4)	51 (49.0)	
Sex					0.027
Female	260 (50.7)	176 (54.9)	39 (47.0)	42 (40.4)	
Male	253 (49.3)	147 (45.1)	44 (53.0)	62 (59.6)	
EITB Results					
Positive	324 (63.2)	187 (57.4)	79 (95.2)	58 (55.8)	0.001
GP50	321 (62.6)	184 (56.4)	79 (95.2)	58 (55.8)	
GP42-39	204 (39.8)	86 (26.4)	77 (92.8)	41 (34.9)	
GP24	189 (36.8)	80 (24.5)	77 (92.8)	32 (30.8)	
GP21	56 (10.9)	0 (0.0)	47 (56.6)	9 (8.7)	
GP18	59 (11.5)	0 (0.0)	48 (57.8)	11 (10.6)	
GP14	46 (9.0)	1 (0.3)	36 (43.4)	9 (8.7)	
GP13	71 (3.8)	6 (1.8)	50 (60.2)	15 (14.4)	
					
Num. of bands					
Median (IQR)	1 (3)	1 (2)	6 (4)	1 (3)	0.001
0	189 (36.8)	139 (42.6)	4 (4.8)	46 (44.2)	0.001
1	119 (23.2)	101 (31.0)	2 (2.4)	16 (15.4)	
2	19 (3.7)	9 (2.8)	0 (0.0)	10 (9.6)	
3	108 (21.1)	71 (21.8)	20 (24.1)	17 (16.4)	
4+	78 (15.2)	6 (1.8)	57 (68.7)	15 (14.4)	
Infection outcomes					
Num. of pigs with viable cysts *	106 (20.7)	18 (5.5)	76 (91.6)	12 (11.7)	0.001
Num. of pigs with degenerated cysts *	55 (10.7)	31 (9.5)	13 (15.7)	11 (10.7)	0.271
Num. of pigs with any cysts *	130 (25.4)	39 (12.0)	76 (91.6)	15 (14.6)	0.001
					
Num. of pigs with viable cysts/	106/130	18/39	76/76	12/15	0.001
Num. of pigs with any cysts *	(81.5)	(46.2)	(100.0)	(80.0)	
					
Num. of pigs with degenerated	55/130	31/39	13/76	11/15	0.001
cysts/Num. pigs with any cysts *	(42.3)	(79.5)	(17.1)	(73.3)	
					
Num. of viable cysts per pig ^†^					
Median (IQR)	137 (951)	2 (53)	841 (900)	109 (2131)	0.001
					
Num. of degenerated cysts per pig ^‡^					
Median (IQR))	4 (13)	3 (7)	3 (5)	26 (72)	0.021

Abbreviations: EITB, enzyme-linked immunoelectrotransfer blot; IQR, interquartile range. * Categories are not mutually exclusive; some pigs may be represented in both categories. ^†^ Among pigs with viable cysts. ^‡^ Among pigs with degenerated cysts.

**Table 2:** EITB class description and EITB banding patterns in each class.

Class Description	N	Num. of Reactive Bands	Banding Patterns ^†^	*n* (%)
Class 1 EITB-negative or only positive for the GP50 band (GP50 antigen family)	308	0 1	0000000 1000000	189 (61.4) 119 (38.6)
Class 2 Positive for antigens of the GP50 family (GP50 band), and the T24/42 family (GP42-39 and GP24 bands), but negative for 8-kDa antigens (GP21, GP18, GP14, and GP13)	127	2 2 3	0110000 1100000 1110000	3 (2.4) 16 (12.6) 108 (85.0)
Class 3 Positive for the GP50 and T24/42 antigen families, as well as GP13 (8-kDa antigen family), and at least positive for 8-kDa antigens GP18 and GP14	22	4 5 5	1110001 1110011 1110101	17 (77.3) 2 (9.1) 3 (13.6)
Class 4 Positive for the GP50 and T24/42 antigen families, as well as 8-kDa antigens GP21 and GP18	55	5 6 6 7	1111100 1111101 1111110 1111111	4 (7.3) 8 (14.6) 3 (5.5) 40 (72.7)

^†^ Values of 0 and 1 indicate the absence or presence, respectively, of each of the EITB bands (GP50, GP42-39, GP24, GP21, GP18, GP14, and GP13).

**Table 3:** Bivariate comparisons between pig characteristics and EITB banding pattern classes *.

Characteristics	Class 1	Class 2	Class 3	Class 4	*p*
	*n* = 308	*n* = 127	*n* = 22	*n* = 55	
Study site					0.001
Tumbes	240 (77.9)	80 (63.0)	6 (27.3)	0 (0.0)	
Huancayo	6 (2.0)	20 (15.7)	10 (45.4)	47 (85.4)	
Piura	62 (20.1)	27 (21.3)	6 (27.3)	8 (14.6)	
Age (months)					0.001
Median (IQR)	7 (9)	10 (12)	12 (18)	24 (24)	
≤8 months	181 (58.8)	50 (39.4)	4 (18.2)	2 (3.6)	0.001
>8 months	127 (41.2)	77 (60.6)	18 (81.8)	53 (96.4)	
Sex					
Female	159 (51.6)	69 (54.3)	10 (45.4)	22 (40.0)	0.316
Male	149 (48.4)	58 (45.7)	12 (54.6)	33 (60.0)	
Infection outcomes					
Num. of pigs with viable cysts **	2 (0.7)	35 (27.6)	16 (72.6)	53 (96.4)	0.001
Num. of pigs with degenerated cysts **	13 (4.2)	19 (15.0)	8 (36.4)	15 (27.3)	0.001
Num. of pigs with any cysts **	15 (4.9)	44 (34.7)	17 (77.3)	54 (98.2)	0.001
					
Num. of pigs with viable cysts/	2/15	35/44	16/17	53/54	0.001
Num. of pigs with any cysts **	(13.3)	(79.6)	(94.2)	(98.2)	
					
Num. of pigs with degenerated cysts/	13/15	19/44	8/17	15/54	0.001
Num. of pigs with any cysts **	(86.7)	(43.2)	(47.1)	(27.8)	
					
Num. of viable cysts per pig ^†^					
Median (IQR)	1 (ND)	100 (286)	123 (969)	1000 (900)	0.001
					
Num. of degenerated cysts per pig ^‡^					
Median (IQR)	1 (5)	3 (6)	5 (8)	1 (75)	0.083

Abbreviations: EITB, enzyme-linked immunoelectrotransfer blot; IQR, interquartile range; ND, not determined. * Classes: 1, EITB-negative or only positive for the GP50 band (GP50 antigen family); 2, positive for antigens of the GP50 family (GP50 band), and the T24/42 family (GP42-39 and GP24 bands), but negative for 8-kDa antigens (GP21, GP18, GP14, and GP13); 3, positive for the GP50 and T24/42 antigen families and 8-kDa antigens GP13, GP14, and GP18 but negative for GP21; 4, positive for the GP50 and T24/42 antigen families and 8-kDa antigens GP21 and GP18. ** Categories are not mutually exclusive; some pigs may be represented in both categories. ^†^ Among pigs with viable cysts. ^‡^ Among pigs with degenerated cysts.

**Table 4:** Odds ratios of having viable cysticercosis according to pig characteristics obtained from logistic regression models *.

Characteristics	Bivariate Model		Multivariate Model	
	OR (95% CI)	*p*	OR (95% CI)	*p*
Age (months)				
≤8 months	Ref.		Ref.	
>8 months	15.85 (5.14 to 48.88)	<0.001	6.04 (3.26 to 11.18)	<0.001
Sex				
Female	Ref.			
Male	1.14 (0.87 to 1.50)	0.334		
EITB banding pattern classes **				
Class 1	0.02 (0.00 to 0.10)	<0.001	0.02 (0.00 to 0.14)	<0.001
Class 2	Ref.		Ref.	
Class 3	7.01 (2.29 to 21.49)	0.001	5.63 (1.20 to 26.41)	0.028
Class 4	69.66 (8.60 to 546.39)	<0.001	47.99 (6.75 to 341.01)	<0.001
Degenerated cysts				
Negative	Ref.		Ref.	
Positive	6.58 (1.91 to 22.72)	0.003	2.22 (0.68 to 7.26)	0.187

Abbreviations: EITB, enzyme-linked immunoelectrotransfer blot; OR, odds ratio; CI, confidence interval. * Regression models used cluster-robust variances to account for the correlation of the data by study site. ** Classes: 1, EITB–negative or only positive for the GP50 band (GP50 antigen family); 2, positive for antigens of the GP50 family (GP50 band) and the T24/42 family (GP42-39 and GP24 bands), but negative for 8-kDa antigens (GP21, GP18, GP14, and GP13); 3, positive for the GP50 and T24/42 antigen families and 8-kDa antigens GP13, GP14, and GP18 but negative for GP21; 4, positive for the GP50 and T24/42 antigen families and 8-kDa antigens GP21 and GP18.

## Data Availability

The datasets collected for this study are available from the corresponding author upon request.

## Supplementary Materials

The following supporting information can be downloaded at: https://www.mdpi.com/article/10.3390/pathogens13010015/s1, Table S1: Goodness-of-fit statistics to select the optimal number of classes for EITB banding patterns in latent class analysis; Table S2: Conditional class-membership probabilities for each EITB band and class prevalences obtained in the 4-class model.
