# Supplementary material for: Antibody Banding Patterns on the Enzyme-Linked Immunoelectrotransfer Blot (EITB) Assay Clearly Discriminate Viable Cysticercosis in Naturally Infected Pigs
Source: Pathogens. 2023 Dec 23;13(1):15. doi: 10.3390/pathogens13010015 (PMC10820179; doi:10.3390/pathogens13010015)
Supplement: Supplementary file 1 [file pathogens-13-00015-s001.zip › Supplementary table S1.pdf]

Supplementary table S1. Goodness-of-fit statistics to select the optimal number of classes for EITB banding patterns in latent class analysis.

**Information criteria**	**Nº of latent classes**				
	2	3	4	5	6
Akaike	2216.3	1785.9	1779.1†	1791.8	1806.3
Bayes	2279.9	1883.3†	1901.5	1957.1	2005.5

†Optimal number of latent classes selected according to statistical criteria.
