# Supplementary material for: Antibody Banding Patterns on the Enzyme-Linked Immunoelectrotransfer Blot (EITB) Assay Clearly Discriminate Viable Cysticercosis in Naturally Infected Pigs
Source: Pathogens. 2023 Dec 23;13(1):15. doi: 10.3390/pathogens13010015 (PMC10820179; doi:10.3390/pathogens13010015)
Supplement: Supplementary file 1 [file pathogens-13-00015-s001.zip › Supplementary table S2.pdf]

Supplementary table S2. Conditional class-membership probabilities for each EITB band and class prevalences obtained in the 4-latent class model.

Glycoprotein bands	Classes†			
	1	2	3	4
GP50	0.39	0.98	1.00	1.00
GP42-39	0.00	1.00	1.00	1.00
GP24	0.00	0.87	1.00	1.00
GP21	0.00	0.00	0.00	1.00
GP18	0.00	0.00	0.14	1.00
GP14	0.00	0.00	0.09	0.78
GP13	0.00	0.00	1.00	0.87
Class prevalence	60.2%	24.8%	4.3%	10.7%

†Classes: 1 (EITB-negative or only positive to the GP50 band (GP50 antigen family); 2 (Positive to antigens of the T24/42 families (GP42-39 and GP24 bands), and negative to 8-kDa antigens (GP21, GP18, GP14, and GP13); 3 (Positive to the GP50 and T24/42 antigen families, and positive to 8-kDa antigens GP18, GP14, and GP13, but negative to GP21); 4 (Positive to the GP50 and T24/42 antigen families, and positive to 8-kDa antigens GP21 and GP18).
